# MatchTope: A tool to predict the cross reactivity of peptides complexed with Major Histocompatibility Complex I

**DOI:** 10.3389/fimmu.2022.930590

**Published:** 2022-10-28

**Authors:** Marcus Fabiano de Almeida Mendes, Marcelo de Souza Bragatte, Priscila Vianna, Martiela Vaz de Freitas, Ina Pöhner, Stefan Richter, Rebecca C. Wade, Francisco Mauro Salzano, Gustavo Fioravanti Vieira

**Affiliations:** ^1^ Bioinformatic Core, Immunogenetics Laboratory, Genetics Department, Biosciences Institute, Federal University of Rio Grande do Sul, Porto Alegre, Brazil; ^2^ Molecular and Cellular Modeling Group, Heidelberg Institute for Theoretical Studies (HITS), Heidelberg, Germany; ^3^ Center for Molecular Biology (ZMBH), DKFZ-ZMBH Alliance and Interdisciplinary Center for Scientific Computing (IWR), Heidelberg University, Heidelberg, Germany; ^4^ Post-Graduation Program in Health and Human Development, Universidade La Salle Canoas, Canoas, Brazil

**Keywords:** T-cell, T-cell response, immunoinformatic, cross reactivity, cross reactivity prediction

## Abstract

The therapeutic targeting of the immune system, for example in vaccinology and cancer treatment, is a challenging task and the subject of active research. Several *in silico* tools used for predicting immunogenicity are based on the analysis of peptide sequences binding to the Major Histocompatibility Complex (pMHC). However, few of these bioinformatics tools take into account the pMHC three-dimensional structure. Here, we describe a new bioinformatics tool, MatchTope, developed for predicting peptide similarity, which can trigger cross-reactivity events, by computing and analyzing the electrostatic potentials of pMHC complexes. We validated MatchTope by using previously published data from *in vitro* assays. We thereby demonstrate the strength of MatchTope for similarity prediction between targets derived from several pathogens as well as for indicating possible cross responses between self and tumor peptides. Our results suggest that MatchTope can enhance and speed up future studies in the fields of vaccinology and cancer immunotherapy.

## Introduction

The Immune System (IS) is the primary defense of an organism against a wide range of exogenous pathogens like viruses, bacteria, and fungi, as well as endogenous pathological conditions like tumor cells (1). However, an inadequate immune response to self, healthy cells, or peptides, is not desirable, as it can lead to autoimmune diseases (2). Several cell types and molecules, such as cell receptors, chemokines, and interleukins, are involved in the immune response, and the complex interactions between these components drive the human immune system (3).

The first step for the IS to mount an immune response and defend the organism is to recognize possible harmful pathogens. One of the ways the human IS accomplishes this task is by loading the Major Histocompatibility Complex (MHC) with a peptide (pMHC) (4) and presenting it to immune cells. This presented epitope can be derived from a self-protein, a protein from a pathogen, or a tumor cell protein (5). There are two main MHC types - MHC class I (MHC-I) and MHC class II (MHC-II) - that differ essentially in which cells they are expressed by and by which immune cells they are recognized. The MHC loci are called Human leukocyte antigens (HLA) in humans.

The cells responsible for pMHC interaction are the T lymphocytes. Among the different T lymphocytes subtypes, two subpopulations coordinate the immune response: the CD8+, or cytotoxic T cells, and the CD4+, or helper T cells. While CD4+ binds to MHC-II, which are expressed by Antigen Presenting Cells (APCs), CD8+ binds to MHC-I. The focus of the current work, MHC-I, is virtually expressed by all nucleated cells and is the central player in presenting every peptide produced inside these cells. The presented epitope can be recognized either as self or non-self. If the epitope is recognized as non-self, a signaling cascade will be triggered, leading to the apoptosis of the infected or tumor cell (6). However, this recognition is not strictly specific: The T-cell receptor (TCR) not only recognizes an exact match of the epitope but also similar ones. This latter event is called cross-reactivity (7, 8) and can lead to unwanted immune responses. Expanding recognition broadness has a positive side since it allows a reduction in the number of TCRs required. However, an epitope derived from a virus protein can mimic a self-epitope and thus trigger an autoimmune disease (2, 9). Furthermore, this is a major limitation to the immune response to tumors, given the high similarity between proteins from normal and tumor cells, making appropriate response difficult for CD8+ cells (1, 10, 11).

The triggering of an immune response depends on the protein interaction between the TCR and the pMHC, in which interface complementarity is a pivotal element. Several physicochemical elements govern this event, such as electrostatic potential distribution. Several works have already described its central role in protein interactions in intracellular and extracellular environments (12, 13). However, beyond that, the charge complementary has an additional function: to guide the anchoring of the protein interaction system more than any other factors (14).

Cross-reactivity becomes particularly important in vaccine development. It is crucial to check whether the vaccine will be effective against all subtypes of a given pathogen (as in the case of dengue viruses, where cross-reactivity between subtypes can lead to hemorrhagic fever) (15). Likewise, when developing a new immunotherapeutic approach, it is necessary to ensure that the target will not trigger cross-reactivity with a self-protein. Given that testing all possible pMHCs *in vitro* is impossible, *in silico* analyses can be helpful. Some cross-reactivity predictors are available, mainly using linear peptide sequences as input, and were primarily designed to predict allergic processes (16–18). However, it is already known that some epitopes show cross-response despite sharing fewer than 50% of amino acid residues in their linear sequence, which implies substantial difficulties for such predictors to predict cross-reactivity correctly (19, 20). For this reason, we developed a new cross-reactivity prediction tool, the MatchTope, which uses protein structural information to predict similarities between pMHC-I complexes, facilitating the development of new vaccines and immunotherapies. Using several available datasets, we verified that MatchTope achieves excellent agreement with experimental results, indicating that this tool can significantly improve vaccine development for several diseases and cancer immunotherapeutic treatments.

## Results

### Opening the MatchTope black box

The MatchTope tool uses the calculation of molecular electrostatic potentials (MEP) of MHC class I loaded with different peptides, followed by clustering the different peptide-MHC class I (pMHC) complexes based on their MEPs similarity. The application of MEP differences as a measure of pMHC class I similarity was previously described by our research team (21, 22).

**Figure 1:**
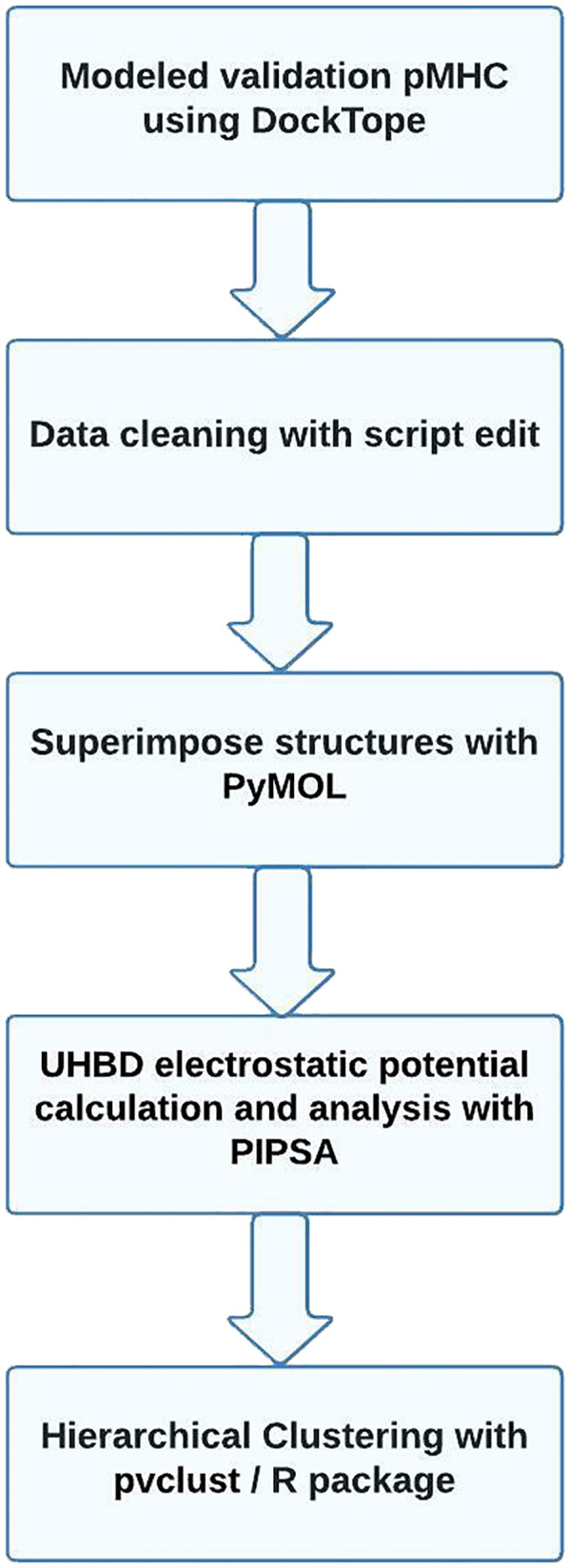
MatchTope flowchart showing the analysis process from the first step of inputting pdb files to the final step of generating results. Each step is described in greater detail in the Methods section.

The steps involved in our analysis are displayed in **Figure 1**. Prior to the analysis, the user should provide a set of pMHC class I files in PDB format (23) (a minimum of three files are required). Since only few crystallographic complexes exist to date, the input pdb file will often stem from a modeling approach. The pdb file contains three columns holding the 3D coordinates of each protein atom as well as some additional information, such as occupancy, temperature factor, element name, charge, radius, or other properties, depending on the source. Since some columns of non-standard pdb files for modeled complexes were found to cause problems during the MEP calculation, these were deleted in a pre-processing step using a bash script.

The next step involves a repositioning of the 3D orientation of all input complexes by superimposing them. This process is important to ensure the comparison of the same electrostatic regions in different pMHCs. To achieve this, we use a Python script to call the PyMOL ‘Fitting’ function (24). This function superimposes the pdb input with a predefined model pMHC pdb structure to unify input positions.

**Figure 2:**
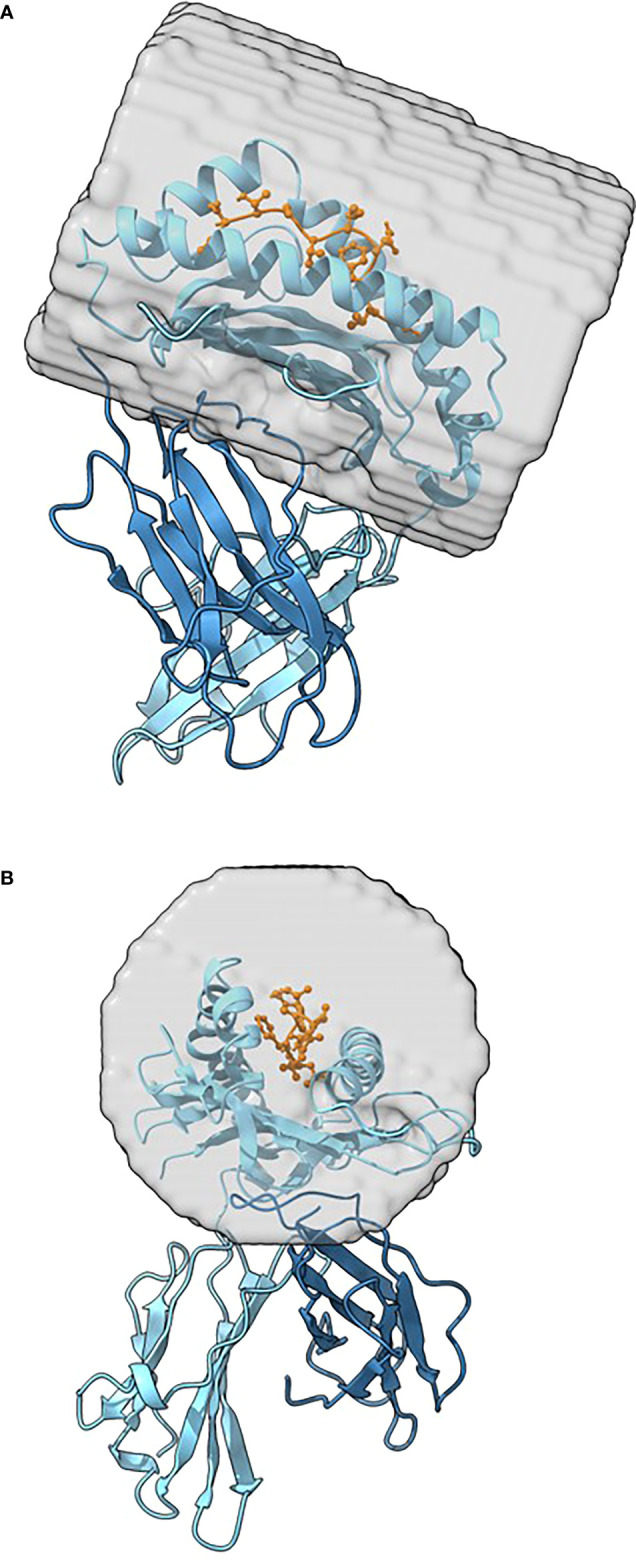
Two different views of a pMHC showing the exact region and size of the cylindrical region used by PIPSA to compare MEPs between different pMHCs. The pMHC is shown in the representation with the alpha chain in light blue color, the beta chain in dark blue color, the epitope in orange with amino acid side chains in stick representation. The cylindrical region used for calculation is shown by a gray semi-transparent surface. The pMHC is shown from the top **(A)** and from the side **(B)**. The pMHC depicted was modeled with the DockTope (28) tool using a dengue virus epitope as input.

After the fitting process, MatchTope starts to calculate the electrostatic similarity of the complexes by using the standalone version of PIPSA. The PIPSA (Protein Interaction Property Similarity Analysis) software is an established tool for analyzing protein electrostatic interaction similarities (https://pipsa.h-its.org/pipsa/) (25, 26). We added modifications to PIPSA to adapt it for the pMHC analysis, accounting for the typical elongated shape of the pMHC binding cleft, which differed from the globular protein shape PIPSA has largely been previously applied to; these modifications are available in PIPSA version 3.2 or later. PIPSA first calculates the MEP using the University of Houston Brownian Dynamics (UHBD) program (27). PIPSA creates a ‘skin’ around each pMHC and then the MEPs of each pMHC complex are compared. Besides calculating overall electrostatic similarities for the full proteins in the complete skins, the algorithm also allows for calculating similarities in a focused region. For this study, a cylinder in the pMHC cleft was considered, and only regions of protein skins residing within this cylinder were used for computing similarity indices, as shown in **Figure 2**. Using this focused region, we can reduce the noise caused by identical surroundings, and thereby avoid erroneous clustering of the results.

The final part of the analysis, the clustering process, uses the similarity indices calculated during the PIPSA run as input. To group electrostatically similar pMHCs in the same cluster, MatchTope uses an R (29) package called ‘pvclust’ (30), which performs a hierarchical clustering combined with a bootstrap of the input data to validate the clustering branch. The cluster package requires some user-defined arguments. We used the “correlation method” to calculate the distance between branches, and the “complete method” as the cluster method. After testing all other criteria, these two arguments yielded the best correlation with the *in vitro* results.

### MatchTope validation

To validate MatchTope, we used four data sets which were obtained from previously published articles (15, 31–33). A list of all considered epitopes, stating also which of them trigger *in vitro* cross-reactivity, is shown in **Supplementary Table 1**, and data on input superposition and model pdb structures is shown in **Supplementary Table 2**. The low average RMSD obtained (0.019 Angströms, considering all protein atoms) indicates that all MHC structures were well superimposed.

**Figure 3:**
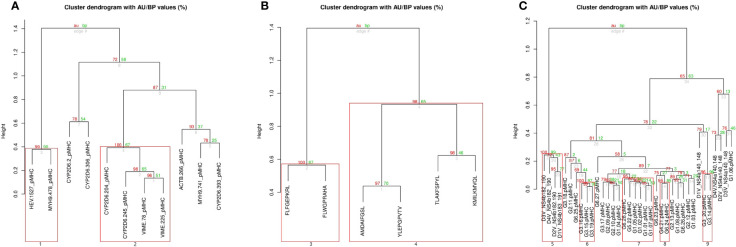
The final result of MatchTope analysis: Hierarchical clustering is represented in a tree-based format. Pvclust provides two types of p-values: AU (Approximately Unbiased, red color) p-value and BP (Bootstrap Probability, green color) value. Branches inside red squares have an AU p-value higher than 95%, indicating a significant similarity between pMHCs clustered in that branch. There are three different clusters matching the different data sets used for MatchTope validation. **(A)** is related to the Hepatitis E Virus (HEV)-Specific T Cell Receptor study, **(B)** is the cluster from the throat cancer study, and **(C)** combines the clustering from the HCV and Dengue studies. Groups 1, 3, 5, 7, and 8 clustered epitopes that have cross-reactivity confirmed by in vitro data. **Supplementary Table 1** presents the epitope list and which epitopes similarly trigger cross reactivity.

The first data set used for MatchTope validation was from a study testing a Hepatitis E Virus (HEV)-Specific T Cell Receptor against some epitopes derived from RNA-dependent RNA polymerase (HEV.1527), non-muscle Myosin Heavy Chain 9 (MYH9.478) and from other proteins (33). The *in vitro* assays show cross-reactivity between HEV.1527 and MYH9.478 and a non-cross recognition between HEV.1527 and ACTB.266. **Figure 3** presents the results obtained with MatchTope during the validation process. Cluster letter A depicts two groupings: 1 and 2. Grouping 1 clustered HEV.1527 and MYH9.478, matching with *the in vitro* results while also putting ACTB.266 on the most distant branch from grouping 1. Grouping 2 clustered different epitopes, but no experimental information regarding potential cross-reactivity was available in the original publication.

In the second data set, six epitopes derived from throat cancer (31) were used. In this study, two major clusters of epitopes are presented, which trigger responses from different TCRs. Within each cluster, the epitopes trigger the response from the same TCR. Our results corroborate the same grouping pattern observed in *in vitro* assays. In cluster letter B (**Figure 3**), it is possible to observe two major groupings – 3 and 4 – which each cluster the epitopes triggering responses from the same TCR.

The third data set used for MatchTope validation was from a Hepatitis C target cross-reactivity study (32). In this study, 28 epitopes presented to HLA-A*02-01 were tested against a wild type viral epitope. Results from *in vitro* experiments demonstrated a cross-reactivity between epitopes from wild type virus and epitopes from genotypes I, IV, V, and VI, which were recognized, fully or partially, by the same TCR recognizing also the wildtype viral epitope. The fourth data set that we have chosen for MatchTope validation was from a study on Dengue virus (15). In this data set, eight pMHCs containing peptides derived from two different proteins, NS4a and NS4b, from the four dengue virus serotypes, are considered. *In vitro* data showed that epitopes generated from NS4b presented cross-reactivity with all other epitopes, while for epitopes generated from NS4a the same was not true. DockTope was first used to model the complexes and then MatchTope was used to compute their MEP similarity. The letter C cluster in **Figure 3** depicts results from both the Hepatitis C (HCV) and Dengue data sets. In this cluster, it is possible to observe that the N4Sb epitopes were placed together (Grouping 5), indicating similarity between targets, which explains the cross-reactivity shown in the *in vitro* assays. Moreover, the NS4a epitopes were not clustered, which corroborates the *in vitro* results. The 6, 7, 8 and 9 grouping from Cluster C are related to HCV data. Previous experiments performed *in vitro* already demonstrated the cross-reactivity indicated here by the 7 and 8 grouping, supporting the efficiency of our tool in grouping targets correctly. Despite the absence of *in vitro* analyses supporting the cross-reactivity predicted in 6 and 9 groupings, it is quite likely that members of these groups would trigger cross-reactivity if tested against a specific TCR.

Additionally, we used a set of distinct complexes studied *in vitro* and deposited in the CrossTope data bank (http://crosstope.com/) (34), for which cross-reactivity has not yet been demonstrated experimentally. In CrossTope, MEP superimposed onto pMHCs molecular surfaces are available, allowing the detection of similar patterns and manual grouping of pMHCs into various clusters based on these similarities. The automated MatchTope analysis, again, led to the expected result with pMHCs with a similar electrostatic charge distribution clustering in the same group (data not shown).

To obtain the best parameters to match *in silico* and *in vitro* results, different combinations of PIPSA settings, as well as statistical parameterization, were tested. For statistical analysis, we tested several clustering options. For PIPSA, we varied probe size, skin thickness, and the cylindrical shape radius by which the focused region is defined. We arrived at a cylindrical shape of 40 Å radius, 33 Å length, 25 Å skin and a probe size of 1 Å. The cylinder is placed on the pMHC cleft, by using the coordinates of the input pdb files as a reference and, with the help of visual analysis, entering those coordinates in the PIPSA settings, as also shown in **Figure 2**. The pMHC pdb file with the cylinder can then be exported to a separate file.

### MatchTope availability

Upon publication of this article, a standalone version of MatchTope will be made available for download, free of charge, *via* github (https://github.com/Marcus-Mendes/MatchTope.git). Also, in the near future, we will release a MatchTope web server version, where pdb files or complexes modeled using DockTope can be uploaded and then directly subjected to MatchTope analysis. The results will then be displayed on the web page and be available for download.

## Discussion

Here, we described a fully automated tool for comparing and clustering pMHCs by MEP similarity for cross-reactivity prediction. Using previously published data sets as input, we were able to correctly group the targets showing cross-reactivity. MatchTope allows the user to analyze multiple pMHC structures at once, calculate the MEPs, and group similar complexes. The resulting distances in molecular electrostatic potential space enable the user to draw conclusions about whether cross-reactivity is likely to occur for the analyzed complexes or not.

The current implementation of MatchTope makes use of various bash scripts, R scripts, and version 4.0.2 of PIPSA. In addition, as an external tool, PyMOL is required. We recommend using the DockTope tool for modeling targets (28). With this tool, the user can model peptides complexed with HLA-A*02:01, HLA-B*27:05, H2-Db, or H2-Kb, but any pMHC of class I allele can be used as input for MatchTope. MEPs are always calculated with the same settings, even if the pMHC allele differs between different complexes, making the MatchTope applicable for other MHC Alleles.

In a previous study (22), we discussed how one of the TCR variable domains, CDR3, discriminates peptides from a self or non-self-protein. In our new tool MatchTope, we solely make use of these regions to calculate MEP similarities. Other portions of the complex were not considered since it would only increase the noise in the analysis. We plan to include topographic features combined with MEP data in a future MatchTope implementation to further improve analysis robustness.

In cluster C (**Figure 3**), the 7 and 8 groups are separated. However, *in vitro* data indicate that both trigger the same TCR, which also recognizes the wild-type epitope. It would be expected that both clusters would be fused in one cluster, but TCR recognition is not a binary process, with different TCRs presenting divergent requirements of stimulation, which can explain why there are two groups instead of one large one.

Cluster A has groups 1 and 2 (**Figure 3**). Group A matches *in vitro* data (33), while there is no *in vitro* evidence to support a cross-reactivity between epitopes from Group 2. Such a result is important to guide wet lab researchers in suggesting new epitopes that can be used in future assays.

The similar performance yielded by MatchTope, compared to our other approaches to cross-reactivity prediction, raises an important question: why develop and use MatchTope? The former approaches were highly dependent on manual intervention. The recovery of RGB information from the interaction surfaces of pMHCs and their subsequent inclusion in a hierarchical clustering approach demands labor-intensive work, even for a small set of structures, in addition to being error-prone. Besides, electrostatic information from all peptide atoms, plus surrounding cleft regions, avoids that differential variable region may be lost in the analysis.

In our database, CrossTope (http://www.crosstope.com/) (34), hundreds of immunogenic pMHC models are available, for which a pdb file can be downloaded and images of MEPs can be viewed. It has previously been observed by manually comparing images that these immunogenic pMHCs show common patterns of electrostatic charge distributions. With MatchTope, however, a comparison on a much larger scale becomes feasible. MatchTope was able to pinpoint similarities between immunogenic targets which were not previously observed, and thus may be helpful in the field of reverse vaccine development.

The field of cancer immunology is rapidly developing and immunotherapeutic approaches are becoming more and more common and show promising results. One methodology makes use of TCR modifications to enhance affinity against tumor-specific peptides (1, 10). However, one major risk of using this approach is the cross-reactivity with normal cells presenting self-peptides. A well-known case (35) is the cross-reactivity between the melanoma-associated antigen MAGE-A3 and a titin-derived antigen expressed by healthy cardiac cells, which led to the death of two patients. These two peptides have a low sequence similarity, sharing just 5 amino acid residues out of 9, but X-ray crystallography showed structural similarity between them. For such cases, MatchTope can be beneficial as it is able to demonstrate the similarity of the peptides in question without the use of crystallographic data, using only modelled pMHC pdb files. This underlines that MatchTope is a powerful tool to predict such undesirable cross-reactivity.

Together with our validation, these data demonstrate that similarity between pMHCs can be predicted from the structure alone, based on the MEP in the cleft region. Since electrostatic similarity can trigger cross-reactivity events, our tool can be used as a cross-reactivity predictor, overcoming inherent issues in predictors that just use linear sequences as input. Even with low sequence similarity in pMHC structures, *e.g.* less than 50% shared amino acid residues of epitopes, our tool was found to be able to properly cluster the targets as indicated by *in vitro* results and thus seems less prone to yield erroneous classifications than tools based solely on sequence comparison. Thus, MatchTope demonstrates itself as a powerful tool to predict similarities between pMHCs and, thereby, indirectly provides an estimate of the likelihood of a cross-reactivity response.

MatchTope can be used in support of vaccine development in many applications beyond those that we presented here. In the light of the current pandemic caused by the SARS-CoV-2 (36, 37), we find ourselves in a rush to find a vaccine or treatment option to reduce the number of infections and death counts (38). Amongst the many studies aiming at the discovery of novel vaccine targets, some point to the possible cross-response between antigens from coronaviruses (39, 40) and other viruses or even bacteria (41, 42). The relevance of T cells to vaccine development lead us to believe that MatchTope could, for instance, be utilized to improve the efficiency of finding epitopes with notable similarity to known immunogenic targets or for the discovery of possible new epitopes that could be tested as vaccine candidates in reduced time frames.

## Materials and methods

### MatchTope automation

MatchTope is a software to seek similarities in the MEP of pMHCs and to group similar MEP patterns by hierarchical clustering. To do this, we developed a workflow involving 3 bash scripts, a Python script, the PyMOL program (24), the PIPSA standalone version (25), version 4.02 available at: https://pipsa.h-its.org and two R packages, to perform the following steps: (i) to edit pdb files to remove unnecessary columns; (ii) to superimpose all pdb files; (iii) to use these pdb files as input for PIPSA to calculate MEPs and corresponding similarity indices; (iv) to use PIPSA results as input for the R package to perform hierarchical clustering. The MatchTope tool was tested on Linux Ubuntu 14.04 and Ubuntu 16.04 systems. The average run time of MatchTope for a 30 pdb file input is 8 minutes on an Intel core i5-750, 6 GB of RAM. The current implementation further was tested successfully with up to 100 pMHCs as input.

### Pre-processing input pdb files

Since pMHC pdb files are often the output of modeling software, they typically have some columns with unnecessary information, which can cause problems for the PIPSA software. To avoid any issues, a bash script removes these columns using shell instructions. After this process, the pdb files retain nine columns, namely the ATOM or HETATM identifier for proteins and other groups, respectively, atom number, type of atom, the corresponding amino acid residue, chain information, amino acid residue number and the Cartesian x-, y- and z coordinates.

### Fitting

To avoid the problem of comparing different regions of different pMHCs due to a nonuniform orientation in the 3D space, we implemented a fitting routine in a Python script making use of the PyMOL software (24). We employ a model PDB distributed along with the code to define the reference position and all input structures are fitted to this model. The script repeats this superposition process twice to ensure a good result.

### PIPSA calculation

PIPSA first computes similarity indices for the electrostatic potential analytically from pdb files, making use of monopole and dipole terms. Hydrogen atoms are added using WHATIF (https://swift.cmbi.umcn.nl/servers/html/index.html) as necessary. Next, the input for UHBD calculations is generated and the electrostatic potential grids computed with UHBD. The PIPSA program then computes the Hodgkin similarity index for all pairs of electrostatic potential grids (25, 43). This is done on the molecular skin and within a cylindrical region of 40 Angström radius and 33 Angström length, defined to encompass the pMHC cleft (using its 3D coordinates derived with the help of visual analysis, as explained before). Due to the fitting step, every PDB file is superimposed, avoiding the comparison of MEPs from different regions. The ‘skin’ represents the remaining layer, after excluding any region inside the solvent-accessible surface area defined with a certain probe radius, and a defined thickness. Everything outside this region is also excluded. Corresponding points on the potential grids within the skins of the two proteins to be compared are used for computing similarity indices. Potential values lying outside of this skin or outside the cylinder created in the pMHCs region of interest will not be used. The thickness of skin and probe radius are adjusted to 25 and 1 Angström, respectively. These non-standard parameters were chosen for the system on the basis of extensive comparison of results obtained using different parameters (**Supplementary Figure 1**). Images were generated with UCSF Chimera 1.12 (44).

### Hierarchical clustering

Once the PIPSA calculation has finished, the program uses the resulting MEP similarities as input to the R package. Using the ‘pvclust’ package, R creates a hierarchical clustering of the results, grouping most similar pMHCs in the same cluster and validating this cluster using bootstrap calculation. The package uses the correlation distance as a metric and complete clustering as the cluster method.

### Validation methodology

To validate our tool, we used four distinct data sets. All targets were nonamers and modeled using the DockTope software (28). A list of all epitope sequences used in our validation step is shown in **Supplementary Table 1**. We modeled all epitopes in HLA*A-0201 complex options, using standard settings. We used the given interferon-gamma results from published data sets to determine cross-reactivity and confirm the validity of our *in silico* analysis (15, 32). The interferon-gamma information and details on individual cutoffs is available in the respective articles.

## Data availability statement

The datasets presented in this study can be found in online repositories. The names of the repository/repositories and accession number(s) can be found below: https://github.com/Marcus-Mendes/MatchTope.

## Author contributions

MM, MB, MF, and GV conceived the study. MM wrote the paper. MM developed the MatchTope scripts. SR modified PIPSA for the analysis of MHC-peptide complexes. SR and RW advised on the use of PIPSA in MatchTope. MM, MF, and MB conducted experiments. MM, MF, IP, GV, and FS analyzed and interpreted the data. IP, GV, RW, PV, and FS were responsible for the general revision of the manuscript. All authors contributed to the article and approved the submitted version.

## Funding

This work was supported by Conselho Nacional de Desenvolvimento Científico e Tecnológico (CNPq) and Coordenação de Aperfeiçoamento de Pessoal de Nível Superior (CAPES). Authors RCW, SR and IP gratefully acknowledge the support of the Klaus Tschira Foundation.

## Acknowledgments

We thank Aruã Ramos Metello de Assis, Mauricio Meneghatti Rigo, and Dinler Amaral Antunes for their involvement in this project.

## Conflict of interest

The authors declare that the research was conducted in the absence of any commercial or financial relationships that could be construed as a potential conflict of interest.

## Publisher’s note

All claims expressed in this article are solely those of the authors and do not necessarily represent those of their affiliated organizations, or those of the publisher, the editors and the reviewers. Any product that may be evaluated in this article, or claim that may be made by its manufacturer, is not guaranteed or endorsed by the publisher.

## Supplementary material

Supplementary Table 1 List of MHC peptide linear sequences and their respective names. Cells of the same color indicate the existence of *in vitro* data demonstrating some level of cross-reactivity event.

Supplementary Table 2 RMSD values (in Angström) of pMHCs used for MatchTope validation compared to the pdb model after fitting. The low RMSD values shown here (below 0.1 Angström) demonstrate that all pMHCs are in the same position. The last row presents the overall mean for all results.

Supplementary Figure 1 Hierarchical clustering from HCV and Dengue studies data using PIPSA’s default values for the cylindrical region, the thickness of skin, and probe radius.
