# Supplementary figures and images for: MatchTope: A tool to predict the cross reactivity of peptides complexed with Major Histocompatibility Complex I
Source: Front Immunol. 2022 Oct 28;13:930590. doi: 10.3389/fimmu.2022.930590 (PMC9650389; doi:10.3389/fimmu.2022.930590)

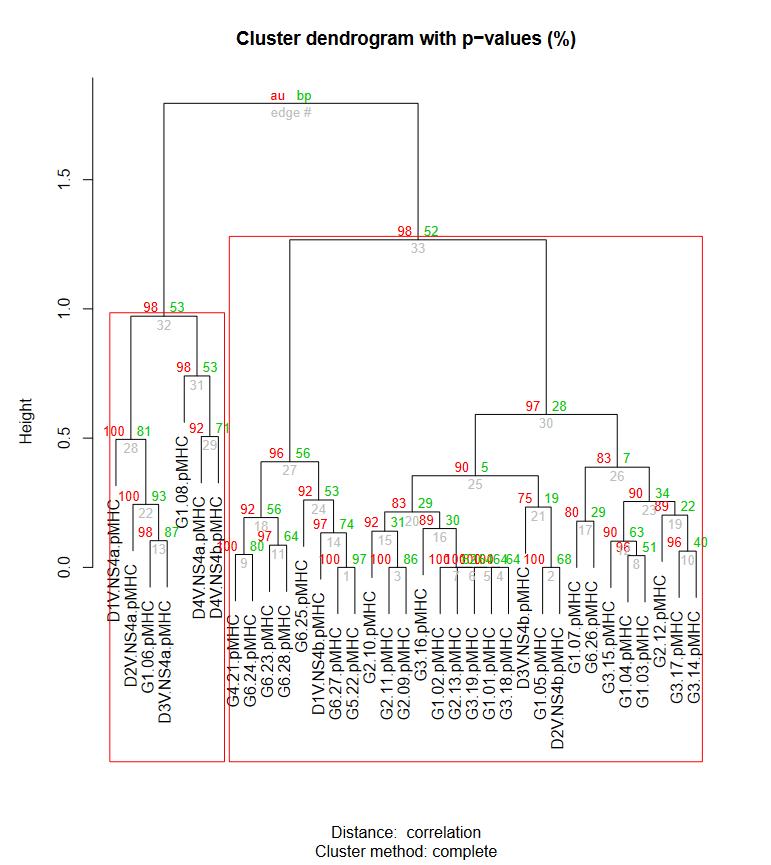

Supplement: Supplementary Figure 1 — Hierarchical clustering from HCV and Dengue studies data using PIPSA’s default values for the cylindrical region, the thickness of skin, and probe radius. [file Image_1.tif]
